# Association between maternal folate concentrations during pregnancy and insulin resistance in Indian children

**DOI:** 10.1007/s00125-013-3086-7

**Published:** 2013-10-26

**Authors:** Ghattu V. Krishnaveni, Sargoor R. Veena, Samuel C. Karat, Chittaranjan S. Yajnik, Caroline H. D. Fall

**Affiliations:** 1 Epidemiology Research Unit, CSI Holdsworth Memorial Hospital, P.O. Box 38, Mandi Mohalla, Mysore 570021 India; 2 Diabetes Unit, KEM Hospital, Pune, India; 3 MRC Lifecourse Epidemiology Unit, Southampton General Hospital, Southampton, UK

**Keywords:** Child, Folate, Homocysteine, Insulin resistance, Pregnancy, Programming, Vitamin B12

## Abstract

**Aims/hypothesis:**

In an Indian birth cohort, higher maternal homocysteine concentration in pregnancy was associated with lower birthweight of the offspring. Lower maternal vitamin B12 and higher folate concentrations were associated with higher offspring insulin resistance. Disordered one-carbon metabolism during early development may increase later metabolic risk. We explored these associations in another birth cohort in India at three age points.

**Methods:**

We measured plasma vitamin B12, folate and homocysteine concentrations at 30 ± 2 weeks’ gestation in 654 women who delivered at one hospital. Neonatal anthropometry was recorded, and the children’s glucose and insulin concentrations were measured at 5, 9.5 and 13.5 years of age. Insulin resistance was estimated using HOMA of insulin resistance (HOMA-IR).

**Results:**

Maternal homocysteine concentrations were inversely associated with all neonatal anthropometric measurements (*p* < 0.05), and positively associated with glucose concentrations in the children at 5 (30 min; *p* = 0.007) and 9.5 years of age (120 min; *p* = 0.02). Higher maternal folate concentrations were associated with higher HOMA-IR in the children at 9.5 (*p* = 0.03) and 13.5 years of age (*p* = 0.03). Maternal vitamin B12 concentrations were unrelated to offspring outcomes.

**Conclusions/interpretation:**

Maternal vitamin B12 status did not predict insulin resistance in our cohort. However, associations of maternal homocysteine and folate concentrations with birth size, and with childhood insulin resistance and glycaemia in the offspring, suggest a role for nutritionally driven disturbances in one-carbon metabolism in fetal programming of diabetes.

**Electronic supplementary material:**

The online version of this article (doi:10.1007/s00125-013-3086-7) contains peer-reviewed but unedited supplementary material, which is available to authorised users.

## Introduction

Nutrition during fetal development has long-term health consequences [1]. Fetal nutrition is influenced by maternal nutritional status, and experiments in animal models have shown that both maternal undernutrition and overnutrition can cause obesity, insulin resistance and diabetes in the adult offspring [2, 3]. Vitamin B12 and folate are important nutrients required for nucleic acid synthesis, DNA methylation and cellular growth and differentiation. Several studies have reported that low maternal folate and vitamin B12 concentrations and high homocysteine concentrations (a marker of deranged one-carbon metabolism) predict smaller newborn size [4–7]. However, data linking maternal diet and nutrient status to long-term offspring outcomes in humans are limited.

Recently it was observed in a rural population in Pune, India, that low maternal vitamin B12 concentrations and high erythrocyte folate concentrations during pregnancy were associated with higher insulin resistance in 6-year-old offspring [8]. In another study, among children in Nepal born to mothers who took part in a randomised controlled trial of micronutrients in pregnancy, maternal vitamin B12 deficiency at baseline was associated with higher offspring insulin resistance at the age of 6–8 years [9], though there was no apparent folate effect.

The Parthenon Study in Mysore, India, is an observational study to investigate maternal and fetal factors related to offspring cardiovascular and diabetes risk [10, 11]. We have previously shown that low maternal vitamin B12 status during pregnancy was related to a higher incidence of gestational diabetes mellitus (GDM) [12]. Maternal GDM was a major predictor of the children’s adiposity and insulin resistance at 9.5 years of age [11]. In the current analysis we aimed to replicate the Pune analysis, to examine whether low maternal vitamin B12 and high plasma folate concentrations predicted higher insulin resistance and other cardiometabolic risk factors in children. Our serial follow-up allowed us to investigate these associations at 5, 9.5 and 13.5 years of age.

## Methods

### Study population

**Fig. 1:**
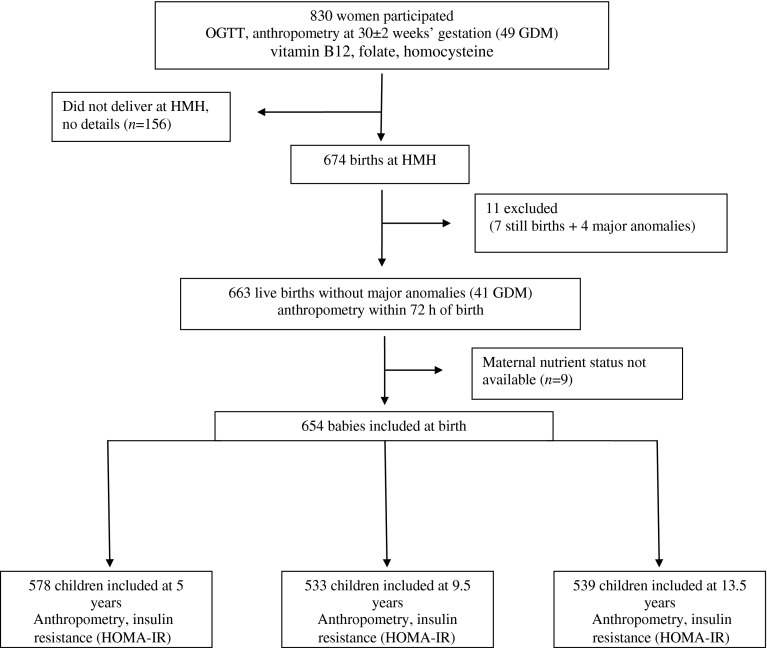
Flow chart of the study participants

During 1997–1998, 830 women booking consecutively into the antenatal clinic of the CSI Holdsworth Memorial Hospital (HMH) in Mysore, India, and matching our eligibility criteria (<32 weeks’ gestation at recruitment, no known history of diabetes, intention to deliver at HMH, singleton pregnancy) had a 100 g, 3 h OGTT at 30 ± 2 weeks’ gestation (Fig. 1) [10]. GDM was diagnosed in 49 women of the 785 women who completed an OGTT (6.2%) using the Carpenter and Coustan criteria [13].

### Vitamin B12 and folic acid supplements

It was routine for general practitioners and obstetricians to prescribe folic acid and/or multivitamin supplements to women throughout pregnancy. Supplement use was recorded at recruitment, but not subsequently, and no information was therefore available on their use at 30 weeks’ gestation, when blood samples were taken, or at term.

### Follow-up of the offspring

At HMH, 663 women delivered live babies without major congenital anomalies. The babies were measured for head and mid–upper arm circumference (MUAC), weight, length, and subscapular and triceps skinfold thicknesses within 72 h of birth as described before [10].

On further follow-up, 25 children had died and eight had major medical conditions. Of the remaining children, detailed anthropometry was performed and percentage body fat (fat%) was measured using bioimpedance in all available children at 5 year (*n* = 585), 9.5 year (*n* = 539) and 13.5 year follow-ups (*n* = 545), and systolic and diastolic BP were measured as described elsewhere [11, 14]. Pubertal status was assessed using Tanner’s method at 9.5 and 13.5 years of age [15], and was classified as the stage of breast development (in girls) or genital development (in boys). The socioeconomic status (SES) of the family was determined at 9.5 years using the Standard of Living Index designed by the National Family Health Survey-2 [16]. Glucose, insulin and fasting lipid concentrations were measured at 5 and 9.5 years from a 2 h OGTT [11, 14].

At 13.5 years of age, glucose, insulin and lipid concentrations were measured using fasting blood samples. The laboratory assays were carried out at the Diabetes Unit, KEM Hospital, Pune, India, whose laboratory is a member of the UK National External Quality Assessment Service (NEQAS) quality control programme for insulin assays. Laboratory staff were blind to the identity of the samples and the vitamin B12 and folate status of the mothers. Plasma glucose and lipid concentrations were measured by standard enzymatic methods (Hitachi-902; Roche Diagnostics, Mannheim, Germany). Insulin was measured by ELISA (Mercodia Ultrasensitive; Mercodia, Uppsala, Sweden). Inter- and intra-assay CV was <7.0%. Insulin resistance was estimated using the HOMA of insulin resistance (HOMA-IR) at all three time points [17].

### Vitamin B12, folate and homocysteine measurements

Maternal vitamin B12, folate and homocysteine concentrations were analysed using plasma samples stored at −80°C for 8 years; adequate samples were available for 654 mothers who were included in the analysis. Measurements were carried out at the Diabetes Unit, KEM Hospital, Pune, using microbiological assays for vitamin B12 and folate and fluorescence polarisation immunoassay (Abbott Laboratories, Abbott Park, IL, USA) for homocysteine [18–20]. The children’s vitamin B12 and folate concentrations were measured at 9.5 years (*n* = 528), and homocysteine concentrations were analysed at 5 years of age (*n* = 567). Intra- and inter-assay CV were <8% for these assays.

Maternal vitamin B12 deficiency was defined as a concentration <150 pmol/l, and folate deficiency a concentration <7 nmol/l. Maternal hyperhomocysteinaemia was defined as a homocysteine concentration >10 μmol/l. These cut-offs were based on the levels used for adult populations elsewhere [21–23].

The HMH research ethics committee approved the study. Informed written consent was obtained from the parents and assent from the children.

### Statistical methods

The distributions of the children’s skinfold thickness, insulin concentrations, HOMA-IR, and vitamin B12 and homocysteine concentrations were skewed; these data were natural log-transformed (ln). Differences between groups were analysed using independent *t* tests. To facilitate comparison of associations, exposure and outcome variables were converted into SD scores (SDS). The data represent SD change in offspring outcome per SD change in maternal nutrient status. Associations between maternal vitamin B12, folate and homocysteine concentrations and offspring outcomes were examined using multivariate linear regression, adjusting for gestational age (for birth variables) or current age (for childhood variables) and sex. Additional adjustments were made for maternal parity, religion, BMI, GDM and SES, and the children’s vitamin B12, folate, current BMI and pubertal stage where relevant; *p* values of <0.05 were considered significant. All analyses were performed using IBM SPSS statistics version 19 (IBM, Armonk, NY, USA).

## Results

Characteristics of the 654 mothers with information on gestational nutrient status, and their children at birth and at 5 (*n* = 578), 9.5 (*n* = 533) and 13.5 years of age (*n* = 539) are presented in Table 1. Vitamin B12 deficiency was present in 41.4% of the women, while only 4% had folate deficiency and hyperhomocysteinaemia. The prevalence of vitamin B12 deficiency was highest among Hindu women (*n* = 183, 49.1%, median vitamin B12 151.0 pmol/l; Muslim women: *n* = 67, 29.8%, 181.0 pmol/l; others: *n* = 21, 37.5%, 180.0 pmol/l). Plasma folate concentrations were lowest among Muslim women (25.8 nmol/l; Hindu women: 40.0 mmol/l; others: 45.7 nmol/l). Homocysteine concentrations were highest among Hindu women (6.3 μmol/l; Muslim women: 5.6 μmol/l; others: 5.6 μmol/l). Maternal homocysteine concentrations were inversely associated with both vitamin B12 (*r* = −0.15, *p* < 0.001) and plasma folate concentrations (*r* = −0.24, *p* < 0.001). Vitamin B12 concentrations were unrelated to SES or parity. Higher SES (*p* < 0.001) and lower parity (*p* < 0.001) were associated with higher plasma folate concentrations. Supplement use at recruitment (mean [SD] 20 [6] weeks’ gestation) was not significantly associated with vitamin B12 and folate concentrations at 30 ± 2 weeks’ gestation.

**Table 1:** General characteristics of the mothers during pregnancy and their offspring

Characteristic	*n*	Value
Mother		
Age, years	654	24.0 (20.0, 26.0)
BMI, kg/m^2^	654	23.3 (21.0, 26.0)
Vitamin B12, pmol/l	654	163.5 (123.0, 224.0)
B12 deficiency, *n* (%)	654	271 (41.4)
Plasma folate, nmol/l	654	35.4 (17.9, 51.2)
Folate deficiency, *n* (%)	654	26 (4.0)
Homocysteine, μmol/l	648	6.0 (5.1, 7.0)
Hyperhomocysteinaemia, *n* (%)	648	25 (3.9)
Offspring		
Birth		
Birthweight, kg	654	2.855 (2.575, 3.165)
Crown–heel length, cm	653	48.7 (47.5, 50.1)
Birthweight SDS	654	−0.9 (−1.6, −0.3)
Birth length SDS	653	−0.5 (−1.1, 0.3)
Head circumference, cm	653	33.9 (32.9, 34.7)
MUAC, cm	651	10.3 (9.6, 10.9)
Subscapular skinfold thickness, mm	651	4.3 (3.9, 4.9)
5 years		
Height, cm	578	105.6 (102.8, 108.3)
BMI, kg/m^2^	578	13.4 (12.9, 14.2)
Height SDS	578	−0.9 (−1.5, −0.3)
BMI SDS	578	−1.4 (−1.9, −0.7)
Insulin resistance (HOMA-IR)	572	0.7 (0.5, 1.1)
Insulin increment	566	26.7 (14.8, 41.9)
9.5 years		
Height, cm	533	130.5 (127.0, 134.4)
BMI, kg/m^2^	533	14.3 (13.4, 15.5)
Height SDS	533	−0.8 (−1.3, −0.2)
BMI SDS	533	−1.3 (−2.0, −0.4 )
Insulin resistance (HOMA-IR)	525	0.8 (0.5, 1.2)
Vitamin B12, pmol /l	522	312.5 (249.8, 404.8)
Plasma folate, nmol /l	522	24.0 (18.0, 35.0)
13.5 years		
Height, cm	539	153.4 (148.9, 158.2)
BMI, kg/m^2^	539	17.0 (15.5, 19.3)
Height SDS	539	−0.7 (−1.4, −0.1)
BMI SDS	539	−0.8 (−1.8, 0.06)
Insulin resistance (HOMA-IR)	528	1.5 (1.0, 2.0)

Values given are median (interquartile range) or *n* (%)

SDSs are based on WHO growth standards

At 9.5 years of age, vitamin B12 deficiency was present in 2.7% of children, and folate deficiency in only one child. Offspring vitamin B12 (*r* = 0.17, *p* < 0.001) and folate concentrations (*r* = 0.19, *p* < 0.001) were positively correlated with maternal vitamin B12 and folate concentrations, respectively. Offspring homocysteine concentrations at 5 years were positively correlated with maternal homocysteine concentrations (*r* = 0.14, *p* < 0.001). Higher maternal folate concentrations were associated with more advanced pubertal stage in the offspring at 13.5 years of age (*p* = 0.04). There was no association between maternal vitamin B12 and homocysteine concentrations and offspring pubertal status.

### Maternal nutrients and neonatal anthropometry

Newborn weight and MUAC tended to increase with maternal plasma folate concentrations, though these associations were markedly attenuated and none were statistically significant following adjustment for maternal covariates (Table 2). Higher maternal homocysteine concentrations were associated with smaller neonatal weight, MUAC and skinfold measurements (Table 2). The prevalence of low birthweight (32.0% vs 19.1%; *p* = 0.1) and preterm (12.0% vs 7.6%; *p* = 0.4) births tended to be higher among those born to mothers with hyperhomocysteinaemia compared with babies born to mothers with normal homocysteine levels. Maternal vitamin B12 concentrations were unrelated to anthropometry at birth (data not shown).

**Table 2:** Mean (SD) anthropometry at birth according to fourths of maternal folate and homocysteine concentrations

Variable	*n*	Weight, kg	Length, cm	Head circumference, cm	MUAC, cm	Subscapular skinfold thickness^a^, mm	Triceps skinfold thickness^a^, mm
Plasma folate concentration, nmol/l							
<17.8	163	2.781 (0.488)	48.3 (2.5)	33.6 (1.6)	10.1 (1.1)	4.4 (3.9, 5.0)	4.0 (3.5, 5.2)
17.9–35.2	163	2.862 (0.472)	48.6 (2.5)	33.7 (1.5)	10.3 (1.0)	4.3 (3.9, 4.9)	4. 1 (3.5, 4.8)
35.3–51.1	164	2.894 (0.492)	48.7 (2.6)	33.7 (1.7)	10.4 (1.0)	4.4 (3.9, 4.9)	4.2 (3.7, 5.7)
>51.1	164	2.900 (0.440)	48.8 (2.0)	33.9 (1.4)	10.3 (1.0)	4.4 (3.9, 4.9)	4.1 (3.6, 4.6)
Model 1							
β (95% CI)		0.07 (−0.007, 0.14)	0.06 (−0.02, 0.14)	0.05 (−0.02, 0.13)	0.08 (0.003, 0.15)	0.01 (−0.07, 0.09)	0.03 (−0.05, 0.11)
*p* value		0.08	0.2	0.2	0.04	0.8	0.5
Model 2^b^							
β (95% CI)		0.02 (−0.06, 0.10)	0.006 (−0.09, 0.10)	0.04 (−0.05, 0.12)	0.02 (−0.06, 0.10)	−0.03 (−0.12, 0.05)	−0.03 (−0.11, 0.06)
*p* value		0.7	0.9	0.4	0.6	0.4	0.6
Homocysteine concentration, μmol/l							
<5.06	163	2.937 (0.446)	48.7 (2.3)	33.9 (1.4)	10.4 (0.9)	4.5 (3.9, 5.0)	4.2 (3.8, 4.9)
5.07–6.01	165	2.876 (0.417)	48.7 (2.2)	33.8 (1.5)	10.4 (1.0)	4.4 (3.9, 4.9)	4.2 (3.7, 4.8)
6.02–7.04	163	2.814 (0.481)	48.5 (2.7)	33.6 (1.7)	10.1 (1.1)	4.3 (3.9, 4.9)	3.9 (3.5, 4.6)
>7.04	163	2.789 (0.539)	48.4 (2.6)	33. 7 (1.6)	10.1 (1.2)	4.3 (3.7, 5.0)	4.0 (3.5, 4.8)
Model 1							
β (95% CI)		−0.12 (−0.19, −0.05)	−0.06 (−0.15, 0.02)	−0.04 (−0.12, 0.04)	−0.12 (−0.19, −0.04)	−0.07 (−0.15, 0.01)	−0.09 (−0.17, −0.01)
*p* value		0.001	0.1	0.3	0.002	0.08	0.03
Model 2							
β (95% CI)		−0.13 (−0.21, −0.05)	−0.09 (−0.18, 0.002)	−0.06 (−0.14, 0.02)	−0.15 (−0.23, −0.07)	−0.12 (−0.20, −0.04)	−0.12 (−0.20, −0.03)
*p* value		0.001	0.06	0.1	<0.001	0.006	0.006

β and *p* values derived by linear regression using maternal folate and homocysteine concentrations, and newborn measurements as continuous SDS; β represents SDS change in outcome variable per SDS change in exposure variable

^a^Log-transformed variables, values given are geometric mean (interquartile range)

Model 1, adjusted for baby’s sex and gestational age

Model 2, model 1 + maternal BMI, GDM status, SES, parity, religion and ^b^vitamin B12

### Maternal nutrients and children’s anthropometry

There were positive associations between maternal folate concentrations and offspring weight, height and MUAC at 5 years of age. These associations were not significant after adjusting for maternal covariates and offspring nutrient status (Table 3). Maternal folate concentrations were positively associated with the children’s anthropometric measurements at 9.5 and 13.5 years, except for subscapular skinfold thickness and fat% (Table 3). The strength of these associations diminished after full adjustment, but remained statistically significant for height (Table 3). There were no associations of maternal homocysteine or vitamin B12 concentrations with the children’s anthropometry.

**Table 3:** Associations between maternal folate concentrations (SDS) and offspring outcomes at 9.5 and 13.5 years

Variable	5 years						9.5 years						13.5 years					
	Model 1			Model 2			Model 1			Model 2			Model 1			Model 2		
	β	95% CI	*p* value	β	95% CI	*p* value	β	95% CI	*p* value	β	95% CI	*p* value	β	95% CI	*p* value	β	95% CI	*p* value
Anthropometry (SDS)																		
Weight	0.13	(0.05, 0.2)	0.001	0.05	(−0.05, 0.1)	0.3	0.17	(0.09, 0.3)	<0.001	0.08	(−0.01, 0.2)	0.08	0.18	(0.09, 0.3)	<0.001	0.08	(−0.003, 0.2)	0.06
Height	0.17	(0.09, 0.3)	<0.001	0.07	(−0.02, 0.2)	0.1	0.21	(0.1, 0.3)	<0.001	0.12	(0.02, 0.2)	0.02	0.22	(0.1, 0.3)	<0.001	0.12	(0.02, 1.2)	0.01
BMI	0.04	(−0.03, 0.1)	0.3	0.02	(−0.08, 0.1)	0.7	0.11	(0.02, 0.2)	0.02	0.05	(−0.04, 0.1)	0.3	0.10	(0.02, 0.2)	0.02	0.05	(−0.04, 0.1)	0.3
MUAC	0.04	(0.02, 0.2)	0.01	0.04	(−0.05, 0.1)	0.4	0.14	(0.05, 0.2)	0.001	0.06	(−0.03, 0.2)	0.2	0.12	(0.03, 0.2)	0.006	0.06	(−0.03, 0.1)	0.2
Subscapular skinfold thickness^a^	−0.02	(−0.1, 0.06)	0.6	−0.02	(−0.1, 0.07)	0.7	0.08	(−0.005, 0.2)	0.07	0.04	(−0.05, 0.1)	0.4	0.06	(−0.02, 0.1)	0.1	0.04	(−0.05, 0.1)	0.3
Fat%	−0.03	(−0.1, 0.04)	0. 4	−0.04	(−0.1, 0.05)	0.4	0.04	(−0.03, 0.1)	0.3	0.009	(−0.07, 0.1)	0.8	0.04	(−0.01, 0.1)	0.09	0.03	(−0.05, 0.1)	0.5
Risk factors (SDS)^b^																		
Glucose_0_	0.07	(−0.01, 0.2)	0.09	0.13	(0.03, 0.2)	0.009	0.05	(−0.04, 0.1)	0.3	0.08	(−0.02, 0.2)	0.1	0.001	(−0.09, 0.09)	0.98	0.03	(−0.07, 0.1)	0.6
Glucose_30_	−0.02	(−0.1, 0.07)	0.7	−0.0001	(−0.005, 0.005)	0.96	0.06	(−0.03, 0.2)	0.2	0.03	(−0.07, 0.1)	0.5	–	–	–	–	–	–
Glucose_120_	−0.03	(−0.1, 0.05)	0.5	−0.0003	(−0.005, 0.005)	0.9	0.03	(−0.06, 0.1)	0.6	−0.01	(−0.1, 0.09)	0.9	–	–	–	–	–	–
Insulin_0_^a^	0.01	(−0.07, 0.09)	0.7	−0.0001	(−0.005, 0.005)	0.97	0.08	(−0.004, 0.2)	0.06	0.09	(0.0003, 0.2)	0.04	0.12	(0.04, 0.2)	0.003	0.10	(0.01, 0.2)	0.02
Insulin_30_^a^	0.03	(−0.05, 0.1)	0.4	−0.00003	(−0.005, 0.005)	0.99	0.12	(0.04, 0.2)	0.004	0.08	(−0.02, 0.2)	0.1	–	–	–	–	–	–
Insulin_120_^a^	0.03	(−0.06, 0.1)	0.5	0.001	(−0.004, 0.006)	0.7	0.01	(−0.07, 0.1)	0.8	−0.03	(−0.1, 0.07)	0.6	–	–	–	–	–	–
HOMA-IR^a^	0.02	(−0.06, 0.1)	0.6	0.02	(−0.08, 0.1)	0.7	0.08	(−0.001, 0.2)	0.053	0.10	(0.01, 0.2)	0.03	0.12	(0.04, 0.2)	0.005	0.10	(−0.01, 0.2)	0.03

β and *p* values derived by linear regression using maternal folate and offspring measurements as continuous SDS; β represents SDS change in outcome variable per SDS change in exposure variable

^a^Log-transformed variables

Model 1, adjusted for child’s sex and age

Model 2, model 1 + maternal BMI, GDM status, SES, parity, religion, vitamin B12 concentration, and children’s 9.5 year vitamin B12 and folate concentrations, and ^b^pubertal stage and current BMI

### Maternal nutrients and children’s insulin resistance and risk factors

At 5 years of age, after adjusting for maternal and offspring covariates including current BMI, maternal folate concentrations were positively associated with fasting glucose concentrations in their offspring (Table 3). Maternal folate concentrations were positively associated with fasting insulin and HOMA-IR in the offspring at both 9.5 and 13.5 years of age, and this association remained significant after full adjustment including maternal vitamin B12 and children’s current BMI and pubertal stage (Table 3). There were also positive associations between maternal folate and offspring 30 min insulin concentrations at 9.5 years of age; however, this association became non-significant after full adjustment (Table 3). There were no differences in the associations between maternal plasma folate and offspring outcomes between children whose mothers had low or normal vitamin B12 concentrations, and there were no significant interactions between maternal folate and vitamin B12 deficiency (electronic supplementary material [ESM] Table 1).

Higher maternal homocysteine concentrations were associated with higher 30 min glucose and insulin concentrations at 5 years of age (Table 4). Higher maternal homocysteine concentrations were associated with higher 120 min glucose concentrations in the children at 9.5 years (Table 4). These associations remained significant after further adjusting for birthweight. There was also a borderline positive association between maternal homocysteine and HOMA-IR at 9.5 years of age. There was no association between maternal homocysteine and offspring HOMA-IR at 13.5 years of age.

**Table 4:** Associations between maternal vitamin B12 and homocysteine concentrations (SDS) and offspring risk factors

Risk factor (SDS)	5 years						9.5 years						13.5 years					
	Model 1			Model 2			Model 1			Model 2			Model 1			Model 2		
	β	95% CI	*p* value	β	95% CI	*p* value	β	95% CI	*p* value	β	95% CI	*p* value	β	95% CI	*p* value	β	95% CI	*p* value
Maternal B12 concentration																		
Glucose_0_	−0.07	(−0.15, 0.01)	0.1	−0.11	(−0.19, −0.02)	0.02	−0.03	(−0.11, 0.06)	0.6	−0.05	(−0.16, 0.06)	0.3	0.006	(−0.08, 0.09)	0.9	−0.02	(−0.12, 0.07)	0.6
Glucose_30_	−0.04	(−0.12, 0.04)	0.4	−0.03	(−0.12, 0.06)	0.5	−0.05	(−0.13, 0.04)	0.3	−0.08	(−0.19, 0.03)	0.1						
Glucose_120_	−0.05	(−0.13, 0.03)	0.2	−0.04	(−0.13, 0.05)	0.4	0.02	(−0.06, 0.10)	0.6	0.02	(−0.09, 0.13)	0.7						
Insulin_0_^a^	−0.02	(−0.10, 0.06)	0.6	−0.02	(−0.11, 0.06)	0.6	0.006	(−0.07, 0.09)	0.9	0.003	(−0.09, 0.10)	0.95	0.009	(−0.07, 0.09)	0.8	0.003	(−0.08, 0.08)	0.9
Insulin_30_^a^	0.01	(−0.07, 0.09)	0.7	0.02	(−0.07, 0.10)	0.7	− 0.02	(−0.10, 0.07)	0.7	−0.06	(−0.16, 0.04)	0.3						
Insulin_120_^a^	−0.02	(−0.10, 0.06)	0.7	−0.01	(−0.10, 0.07)	0.8	0.04	(−0.04, 0.12)	0.3	0.03	(−0.07, 0.1)	0.6						
HOMA−IR^a^	−0.03	(−0.11, 0.05)	0.4	−0.03	(−0.12, 0.05)	0.4	0.003	(−0.08, 0.08)	0.9	−0.004	(−0.10, 0.09)	0.9	0.01	(−0.07, 0.09)	0.8	−0.001	(−0.08, 0.08)	0.99
Maternal homocysteine concentration																		
Glucose_0_	0.03	(−0.06, 0.11)	0.6	0.02	(−0.07, 0.11)	0.7	0.06	(−0.03, 0.15)	0.2	0.08	(−0.007, 0.17)	0.07	0.02	(−0.07, 0.10)	0.7	0.06	(−0.03, 0.15)	0.2
Glucose_30_	0.10	(0.02, 0.19)	0.02	0.17	(0.05, 0.30)	0.007	0.06	(−0.03, 0.15)	0.2	0.06	(−0.03, 0.15)	0.2						
Glucose_120_	0.01	(−0.02, 0.05)	0.5	0.05	(−0.04, 0.14)	0.3	0.10	(0.01, 0.19)	0.03	0.11	(0.02, 0.20)	0.02						
Insulin_0_^a^	0.01	(−0.03, 0.05)	0.6	0.03	(−0.06, 0.12)	0.5	0.07	(−0.10, 0.16)	0.09	0.06	(−0.02, 0.14)	0.1	−0.05	(−0.13, 0.03)	0.2	−0.03	(−0.10, 0.05)	0.5
Insulin_30_^a^	0.07	(−0.01, 0.15)	0.1	0.09	(0.01, 0.18)	0.04	0.01	(−0.08, 0.10)	0.8	0.02	(−0.07, 0.10)	0.7	–	–	–	–	–	–
Insulin_120_^a^	0.04	(−0.05, 0.12)	0.4	0.05	(−0.04, 0.14)	0.2	0.04	(−0.05, 0.13)	0.4	0.05	(−0.03, 0.13)	0.2	–	–	–	–	–	–
HOMA-IR^a^	0.03	(−0.05, 0.11)	0.5	0.04	(−0.05, 0.12)	0.4	0.08	(−0.007, 0.16)	0.07	0.02	(−0.003, 0.04)	0.09	−0.04	(−0.12, 0.04)	0.3	−0.01	(−0.09, 0.06)	0.7

β and *p* values derived by linear regression using maternal vitamin B12 and homocysteine concentrations and offspring measurements as continuous SDS; β represents SDS change in the outcome variable per SDS change in the exposure variable

Model 1, adjusted for child’s sex and age

Model 2, model 1 + maternal BMI, GDM status, SES, parity and religion, vitamin B12 concentrations, and children’s 9.5 year vitamin B12 and folate concentrations, pubertal stage and current BMI

^a^Log-transformed variables

Maternal vitamin B12 concentrations were not associated with any of the offspring risk factors (Table 4). Maternal folate, vitamin B12 and homocysteine concentrations were not associated with offspring blood pressure or fasting lipid concentrations (data not shown).

All associations remained unchanged after excluding the offspring of GDM mothers.

## Discussion

In this well-characterised cohort of urban Indian children, higher maternal homocysteine concentration was associated with smaller size at birth, and higher postload glucose concentrations at 5 and 9.5 years. Higher maternal folate concentration was associated with higher insulin resistance (HOMA-IR) at 9.5 and 13.5 years. There were no associations of maternal vitamin B12 concentration with size at birth or with insulin resistance and other cardiometabolic risk markers.

Earlier studies have observed a high prevalence of vitamin B12 deficiency and elevated levels of homocysteine but not folate deficiency among Indians [4, 8]. Consistent with these findings, more than 40% of our study women were vitamin B12-deficient, while only 4% had low serum folate concentrations. The homocysteine concentrations were, however, lower than those reported in other studies in the Indian subcontinent (Table 5). A recent systematic review and meta-analysis found that higher maternal homocysteine concentration in pregnancy is consistently associated with lower birthweight, in different populations [24]. Overall, it showed a 31 g reduction in birthweight per SD increase in homocysteine, and hyperhomocysteinaemia (>90th percentile) was associated with an OR of 1.25 (95% CI 1.09, 1.44) for a small-for-gestational-age baby. Our results are consistent with this, showing a 60 g fall in birthweight per SD of maternal homocysteine concentration, which was echoed in a reduction in all birth measurements. We did not find associations between maternal vitamin B12 or folate status and newborn size. Evidence from other populations has been inconsistent on this point, especially for vitamin B12. The Pune study showed no association between maternal B12 status and birthweight [8], while another Indian study, from Bangalore, showed a positive association [4]. Several studies, including the Pune study, have shown positive associations between maternal folate status and birthweight [5, 6, 8].

**Table 5:** Comparison of maternal nutrients and offspring outcomes in Mysore and studies from other settings

Variable	Mysore	Pune [8, 42]		Nepal [9, 43]	Bangalore [4]	Pakistan [44]	UK [6]	Netherlands [30]
Maternal measurements								
Age, years	23.9 (4.3)	21.4 (3.5)		19.3 (2.1)	24.6 (4.1)	26.0 (0.5)	27.8 (5.8)	33.3 (30.7, 35.5)
Pregnancy stage at measurement	3rd trimester	2nd trimester	3rd trimester	1st trimester	1st trimester	At delivery	1st trimester	3rd trimester
BMI, kg/m^2^	23.6 (3.6)	18.9 (1.8)	20.5 (1.7)	23.1 (5.4)	22.0 (4.0)	–	–	–
Vitamin B12, pmol/l	187 (100)	151 (80)	143.4 (99)	247 (198)	232 (87)	162 (23, 317)	324 (132)	179 (134, 219)
B12 deficiency (<150 pmol/l), *n* (%)	271 (41.5)	380 (60)	423 (71)	147 (27)	177 (47)	–	–	–
Serum folate, nmol/l	35.5 (19.4)	–	–	17.7 (20.2)	–	14 (2, 91)	–	9.1 (6.1, 16.4)
Erythrocyte folate, nmol/l	–	941 (352)	1048 (408)	–	–	–	418 (178)	–
Folate deficiency (<7 nmol/l), *n* (%)	26 (4.0)	1 (0.2)^a^	1 (0.2)^a^	57 (10.5)^b^	–	–	–	–
Homocysteine, μmol/l	6.4 (2.3)	8.8 (3.1)	9.1 (3.2)	–	–	10.3 (4.3, 23)	–	5.5 (4.5, 6.7)
Hyperhomocysteinaemia (>10 μmol/l), *n* (%)	25 (3.9)	177 (28)	193 (33)	179 (34.1)^c^	–	–		
Newborn measurements								
Birthweight, g	2,854 (473)	2,618 (388)		–	2,850 (450)	–	3,430 (470)	3,425 (3,075, 3,855)
Low birthweight^d^, *n* (%)	129 (19.5)	177 (28)		180 (33)			–	–
Child measurements								
Childhood HOMA-IR [17]	0.8 (0.5, 1.2)	0.81 (0.60)		0.4 (0.2, 0.7)	–	–	–	–
Maternal vitamin B12–newborn size	None	None	None	–	Positive	None	None	None
Maternal folate–newborn size	None	None	Positive	–	None	Positive	Positive	None
Maternal homocysteine–newborn size	Negative	Negative	None	–	–	Negative	–	None
Maternal vitamin B12–child HOMA-IR	None	Negative	None	Negative	–	–	–	–
Maternal folate–child HOMA-IR	Positive	None	Positive	None	–	–	–	–
Maternal homocysteine–child HOMA-IR	Borderline positive	None	None	–	–	–	–	–

Values given are mean (SD), median (interquartile range) or *n* (%)

^a^Erythrocyte folate <283 nmol/l

^b^Serum folate <6.8 nmol/l

^c^Homocysteine concentrations >12 μmol/l

^d^Birthweight <2,500 g

The protective effect of maternal folate repletion against neural tube defects is well known. This has led to the recommendation for folate supplementation in the preconceptional period, and, in a number of countries, folate fortification of staple foods, to achieve wide coverage of mothers in the crucial early stages of pregnancy [25]. Studies linking maternal folate status with long-term outcomes in the children are, however, few and are mainly based on observational data [8, 26–28]. In the only data from a randomised controlled trial, children of women in Nepal who were supplemented with vitamin A plus folic acid from early gestation had less microalbuminuria and a lower prevalence of metabolic syndrome at 6–8 years than children of mothers supplemented with vitamin A alone [29]. Interestingly, these reductions in microalbuminuria and metabolic syndrome were not observed in children of mothers who received the same dose of folic acid as part of a multiple micronutrient supplement. In the UK, observational studies have shown higher bone mineral content in children of mothers with higher dietary folate intakes [26]; in the Pune (India) study, higher bone mineral content and density in children of mothers with higher folate status in pregnancy [27]; and in our study reported here, taller height and better cognitive function [28] in children of mothers with higher folate status.

By contrast, the Pune study linked higher maternal folate status with a potentially adverse outcome: higher childhood insulin resistance [8]. Our study in Mysore has now confirmed this, and, for the first time, showed that this association tracks into early adolescence. This is a small effect: a 1 SD higher maternal folate concentration was associated with a ∼0.09 SD increase in childhood HOMA-IR at both 9.5 and 13.5 years of age in our children. In Pune children, there was a similar, albeit bigger, effect: a 0.18 SD increase in HOMA-IR for a 1 SD increase in erythrocyte folate. In Pune, lower maternal vitamin B12 status was also associated with higher offspring insulin resistance, and the highest mean HOMA-IR was in children of mothers with B12 deficiency and high folate status. The only other study to examine this question is the Nepal randomised trial [9]. This trial showed that maternal vitamin B12 deficiency at baseline was associated with higher HOMA-IR in the children, but there was no association with maternal folate status, and no difference in HOMA-IR between children of mothers who were or were not supplemented with folic acid. In Mysore, there was also an association between higher maternal homocysteine concentration and postload glucose concentration in the children. This was not observed in the Pune study, and it was not investigated in the Nepal study.

These differences between studies may arise from differences in maternal micronutrient status and differences in fetal growth (Table 5). Maternal plasma folate levels in Mysore were considerably higher than those reported in Western studies [30, 31]. A predominantly vegetarian diet among these women, and a tendency to use high-dose folic acid supplements throughout pregnancy, may explain this finding. The prevalence of folate deficiency, though uncommon in all three studies, was relatively higher among the Nepal women [9]. The prevalence of vitamin B12 deficiency (which was extremely high in Pune) was high in all studies but lowest in Nepal (27%). Surprisingly, the prevalence of hyperhomocysteinaemia and mean homocysteine concentrations were much lower in Mysore than in Pune and Nepal, which had a similar prevalence. HOMA-IR values in Mysore and Pune were similar, but lower in Nepal. In the Pune and Nepal studies, it was the vitamin B12 status during early pregnancy which was associated with offspring HOMA-IR; however, there was no association with the late-gestation concentrations, similar to the situation in Mysore. Both in our study and in Pune, offspring HOMA-IR was associated with maternal folate at 28 weeks’ gestation. While optimum micronutrients during the early stages of pregnancy have beneficial effects on fetal development, our findings suggest that exposure to high folate levels in late pregnancy may confer long-term metabolic risk.

The mechanism underlying our findings may be speculative. There is evidence that the balance between folate and vitamin B12 status is important for health. In an elderly population in the USA, higher serum folate concentration presumably due to higher intake, especially in the presence of vitamin B12 deficiency, was associated with impaired cognitive function and anaemia [32]. There is a suggestion that the risk of cancer, cardiovascular disease and neuropsychiatric disorders is increased at both high and low levels of folate status [33]. Enzymes that require folate as a cofactor may be inhibited by high levels of folate [34]. Higher folate in our mothers may reflect higher dietary intake or the ‘folate trap’ phenomenon, considering the low vitamin B12 status. In B12 deficiency, the activity of vitamin B12-dependent methionine synthase is reduced and cellular folate is trapped as inactive 5-methyltetrahydrofolate, which may leak into plasma, thus elevating serum folate levels [35, 36]. Impaired methionine synthesis from homocysteine, and thus protein synthesis, may hinder lean tissue deposition. There may also be effects on DNA synthesis or on DNA methylation that may have long-term effects on gene expression and metabolism [36, 37]. The accumulation of homocysteine in the body may also have adverse outcomes.

Our data do not give definitive evidence for the folate trap as a mechanism: first, because there was no interaction between maternal folate and vitamin B12 in relation to the child’s insulin resistance (the association between maternal folate and insulin resistance in the child was not restricted to children of B12-deficient mothers); and, second, because the inverse association between folate and homocysteine was mainly in mothers with low folate concentrations (ESM Fig. 1). The Pune study findings did not support the folate trap either [8]: although there was an interaction between folate and vitamin B12, the folate measurement in Pune was erythrocyte folate, which does not increase in the folate trap. The folate trap mechanism therefore seems unlikely, though it cannot be ruled out from the type of data that we have. Maternal homocysteine level was relatively low despite a high prevalence of B12 deficiency, which may be related to the action of other B vitamins, such as vitamin B2 and B6, and proteins on homocysteine conversion [23].

In our study the association between maternal folate and offspring HOMA-IR was not present at 5 years of age, but emerged at 9.5 years and persisted through to 13.5 years of age. We have previously shown that the association between maternal vitamin D deficiency [38] and glucose intolerance [11, 14] with offspring HOMA-IR was present at 9.5 years of age, but not at 5 years of age. At younger ages, high beta cell activity in children may mask the insulin resistance.

Our study, taken together with the other studies from India and Nepal, suggests that the folate–vitamin B12–homocysteine pathway may have a role in regulating fetal growth and in the intrauterine programming of later diabetes. The ‘proof of principle’ for this comes from animal studies showing that folate and other methyl donor nutrients in the maternal diet influence adiposity and insulin resistance in the adult offspring and that this is associated with epigenetic changes, for example altered DNA methylation [37, 39]. The initial findings from human studies are complex, and more data, especially from randomised trials of folate and/or vitamin B12 in pregnancy, would be helpful.

A major strength of our study was the measurement of circulating maternal nutrients during pregnancy, which is a more robust indicator of maternal nutritional status than dietary or supplement intake levels. Detailed neonatal and childhood anthropometry, and measurement of an array of cardiovascular risk factors were other strengths. Offspring outcomes were available at three time points during childhood and early adolescence, which allowed us to examine whether the observed associations were persistent. The plasma samples had been stored for 8 years before analysing maternal nutrients. These nutrients are thought to be stable over long periods, however, when frozen at −80°C [40]. A comprehensive range of measurements both during pregnancy in the mother and later follow-up in the offspring enabled relevant adjustments. It is likely that plasma folate levels were influenced by folic acid supplements. Unfortunately, we did not collect data on supplement use at 30 weeks’ gestation when maternal folate status was measured. Supplementation could confound the association between maternal folate concentrations and insulin resistance in the child. For example, if mothers of higher SES took more supplements, their children could also have higher insulin resistance because of greater adiposity. The association was unchanged, however, by adjusting for maternal SES and the child’s BMI.

In conclusion, our study replicates the Pune finding of higher insulin resistance in children born to mothers with higher gestational folate levels. It also showed that this association persists through childhood into adolescence. We did not see an association with maternal vitamin B12 status. In the context of other studies, our study suggests that a deranged maternal one-carbon pathway relates to impaired fetal growth and cardiometabolic risks in later life. These are novel associations which may help to elucidate the complex mechanisms underlying the development of type 2 diabetes. These findings are suggestive of ‘nutrient-mediated teratogenesis’, where micronutrient deficiencies during intrauterine development promote increased risks in the offspring [41], captured under the broad ambit of developmental origins of health and disease. It is not clear whether these small changes observed in relation to maternal B vitamins represent an increased risk of later disease. Follow-up of children in different cohorts and randomised controlled trials could help clarify these issues.

## Electronic supplementary material

Below is the link to the electronic supplementary material.

ESM Table 1 (PDF 81 kb)

ESM Fig. 1 (PDF 214 kb)

## Abbreviations

Fat%: Percentage body fat
GDM: Gestational diabetes mellitus
HMH: Holdsworth Memorial Hospital
HOMA-IR: HOMA of insulin resistance
MUAC: Mid–upper arm circumference
SDS: SD score
SES: Socioeconomic status
