# Supplementary material for: Association between maternal folate concentrations during pregnancy and insulin resistance in Indian children
Source: Diabetologia. 2013 Oct 26;57(1):110–21. doi: 10.1007/s00125-013-3086-7 (PMC3855580; doi:10.1007/s00125-013-3086-7)
Supplement: Supplementary file 1 — (PDF 81 kb) [file 125_2013_3086_MOESM1_ESM.pdf]

ESM Table 1. Associations between maternal folate concentrations (SDS) and offspring outcomes according to maternal vitamin B12 status

	5.years					9.5 years					13.5 years				
	B12 deficiency				p^a	B12 deficiency				p^a	B12 deficiency				p^a
	Yes		No			Yes		No			Yes		No		
	β	*p*	β	*p*		β	*p*	β	*p*		β	*p*	β	*p*	
Risk factors (SDS)															
Glucose^{0}	0.03	0.6	0.18	0.008	0.06	-0.04	0.6	0.13	0.06	0.4	0.02	0.8	0.01	0.9	0.8
Glucose^{30}	0.05	0.6	0.003	0.96	0.8	0.07	0.4	0.03	0.7	0.9					
Glucose^{120}	-0.08	0.3	0.03	0.6	0.3	-0.11	0.2	0.05	0.5	0.2					
Insulin$^{0\ b}$	0.02	0.8	-0.009	0.9	0.98	0.04	0.6	0.11	0.054	0.6	0.16	0.02	0.08	0.2	0.3
Insulin$^{30\ b}$	-0.04	0.6	0.05	0.5	0.4	0.06	0.4	0.08	0.2	0.997					
Insulin$^{120\ b}$	-0.09	0.3	0.11	0.09	0.08	-0.15	0.06	0.03	0.6	0.2					
HOMA-IR ^{b,c}	0.02	0.8	0.02	0.8	0.7	0.03	0.1	0.12	0.4	0.6	0.15	0.07	0.08	0.2	0.4

β and P values derived by linear regression using maternal folate and offspring outcomes as continuous SDS. β represents SDS change in the outcome variable per SDS change in the exposure variable. All analyses adjusted for child's sex and age, maternal BMI, GDM status, SES, parity and religion and children's 9.5 year vitamin B12 and folate concentrations and pubertal stage and current BMI.

[a] *p* for interaction (folate*B12 deficiency); [b] log-transformed variable,
