# Supplementary material for: Association between maternal folate concentrations during pregnancy and insulin resistance in Indian children
Source: Diabetologia. 2013 Oct 26;57(1):110–21. doi: 10.1007/s00125-013-3086-7 (PMC3855580; doi:10.1007/s00125-013-3086-7)

ESM Fig. 1: Correlation between maternal folate and homocycteine concentrations according to maternal vitamin B12 and folate status.

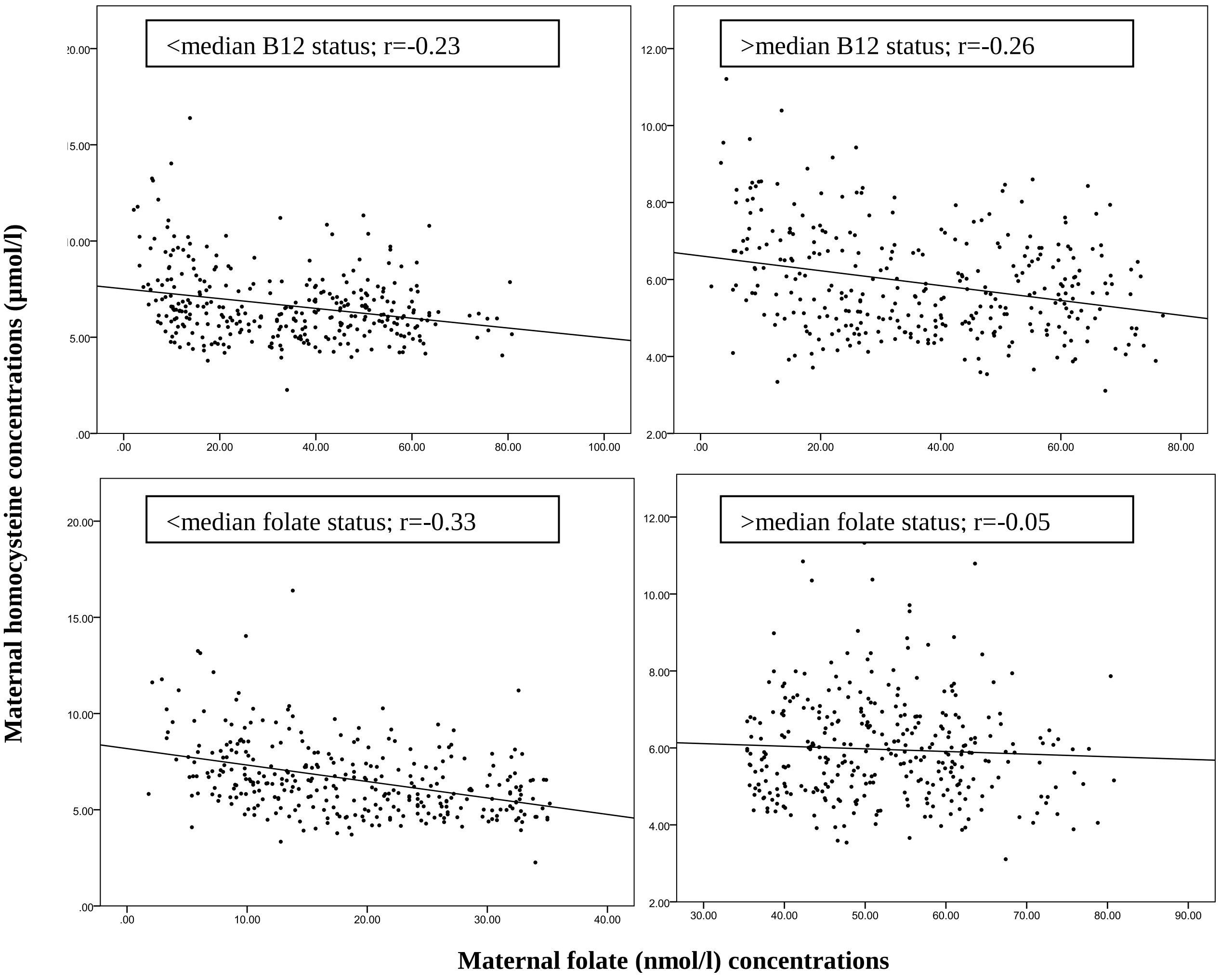

Supplement: Supplementary file 2 — (PDF 214 kb) [file 125_2013_3086_MOESM2_ESM.pdf]
